# Dipyrimadole & Post-Operative Hypercoagulability

**Published:** 1967-01

**Authors:** U. Jayaswal

**Affiliations:** Department of Pathology, Frenchay Hospital


### DIPYRIMADOLE AND POST-OPERATIVE HYPERCOAGULABILITY

BY

U. JAYASWAL

*Department of Pathology, Frenchay Hospital.*

It has been suggested that dipyrimadole might be effective in reducing the incidence of thrombosis by interfering with both platelet clumping and the early stages of blood clotting. Emmons *et al* (1965 a & b) found that dipyrimadole inhibited thrombus formation in injured rabbit vessels *in vivo*, and reduced platelet stickiness *in vitro*, and they suggested that dipyrimadole might be a useful agent in the reduction of thrombo-embolic attacks.

Twenty-seven patients submitted to "cold" surgery at this hospital were treated with either active dipyrimadole or placebo tablets in an attempt to discover whether or not the active substance would be useful in reducing the incidence of post-operative thrombosis, and associated embolism. Two *in vitro* tests were used as indices of the post-operative hypercoagulable state and of any effective drug activity :

(a) the activated plasma clotting time, normally minimal at about the 5th post-operative day;

(b) the adhesive platelet count, normally maximal about the 8-10th post-operative days.

### METHODS AND MATERIALS

Venous blood was collected with siliconed syringes into polystyrene containers before operation, about the 5th day, and about the 8-10th day. The whole blood total platelets and ADP-induced adhesive platelets were counted using a Coulter Counter, Model "A" (Medical) (Eastham 1963, 1964). The plasma activated clotting time, using bentonite as the activating agent and soya bean extract as the platelet substitute, was estimated with an EEL Prothrombinmeter (Eastham 1962).

Patients were roughly paired for age, sex, type of operation; and either the active dipyrimadole or the placebo tablets were given after the first blood sample had been taken, at the rate of 25 mg. four times daily until the 10th post-operative day. The two types of tablets were merely labelled "A" and "B" (i.e., Active and Placebo).

### RESULTS

The activated plasma clotting times found before operation and about the 5th post-operative day are shown in Table I. It will be seen that there is no obvious difference between the two test groups of patients.

TABLE I

| | Drug A (13 cases) | Drug P (14 cases) |
|---|---|---|
| Pre-operative activated clotting time (mean) ... ... ... ... | 34.5 secs. | 34.6 secs. |
| 5th day post-operative activited clotting time (mean) ... ... ... ... | 31.5 secs. | 33.9 secs. |

The whole blood platelet counts, adhesive platelet counts and adhesive platelet counts expressed in relation to the corresponding total platelet counts are shown in Table II. Again, there is no obvious difference between the post-operative adhesive platelet counts in the two groups. The mean initial count and the mean post-operative rise in the platelet count of the two groups likewise do not differ significantly.

TABLE II

| | Drug A (8 cases) | Drug P (14 cases) |
|---|---|---|
| Mean pre-operative total platelet count | 212,880/c.mm. | 221,450/c.mm. |
| Mean post-operative total platelet count 8-10th day ... ... ... | 269,770/c.mm. | 268,830/c.mm. |
| Mean pre-operative adhesive count ... | 86,610/c.mm. | 98,600/c.mm. |
| Mean post-operative adhesive count 8-10th day ... ... ... ... | 132,550/c.mm. | 132,270/c.mm. |
| % adhesive count (pre-operative) ... | 42% | 44% |
| % adhesive count (post-operative) ... | 49% | 50% |

Thus, in this series, dipyrimadole does not appear to affect either the post-operative plasma hypercoagulability or the post-operative increase in ADP-induced adhesive platelets.

Finally, a few of the patients developed severe headaches whilst under treatment with dipyrimadole, which resolved when the drug was discontinued.

### DISCUSSION

Post-operative thrombosis and subsequent embolism are serious causes of ill-health and death. Martin (1966) found that out of 1,500 consecutive post-operative patients, 55 suffered from thromboembolic attacks. After trauma, pulmonary embolism is not uncommon, especially after middle age, and it is a common cause of death in the elderly, arising in half the fatal cases from 'silent' thromboses (Sevitt, 1959). Morrel *et al.* (1963) related the incidence of pulmonary embolism to both the patient's age and the duration of bed rest, and estimated that in two of the hospitals studied there was an average of one preventable death each week. The highest incidence of thrombo-embolic attacks after trauma or surgery occurs in the first two weeks (Sevitt & Gallagher, 1961).

The post-operative rise in both the total platelet count and the adhesive platelet count, and the post-operative shortening of the activated plasma clotting time can be used as a rough *in vitro* measure of hypercoagulability (Eastham 1964; Eastham & Morgan, 1964). However, in this series it was found that dipyrimadole did not affect either the activated plasma time or the adhesive platelet counts when compared with results obtained from similar patients who were given placebo tablets. It remains important to find a non-toxic substance capable of reducing plasma hypercoagulability and the associated increased adhesive platelet count during the few days immediately after operation, as a means of reducing the incidence of thrombosis and associated embolism.

*Acknowledgements :*

I am indebted to Dr. R. D. Eastham for his inspiration, help and encouragement, without which this study would not have been possible, and many thanks are due to Messrs. C. H. Bartlett, H. K. Bourns and T. J. Butler, Consultant Surgeons at Frenchay Hospital, for allowing this study on their patients, and to Bœhringer Ingelheim, Ltd., for supplying the active dipyrimadole and placebo tablets.

### REFERENCES

Eastham, R. D. (1963), *Lancet,* **i,** 53.

Eastham, R. D. (1964), *J. Clin. Path.,* **17**, 275.

Eastham, R. D. (1962), *ibid.,* **15,** 86.

Eastham, R. D. (1963), *J. Clin. Path.,* **16,** 168.

Eastham, R. D. (1964), *Lancet,* **ii,** 541.

Eastham, R. D., and Morgan, E. (1964), *Lancet,* **ii,** 543.

Emmons, P. R., Harrison, M. J. G., and Mitchell, J. R. A. (1965a), *Lancet,* **ii,** 603.

Emmons, P. R., Harrison, M. J. G., Honour, A. J., and Mitchell, J. R. A. (1965b), *Nature,* **208,** 255.

Martin, P. (1966), personal communication.

Morrel, M. T., Truelove, S. C., and Barr, A. (1963), *Brit. Med. J.,* **ii,** 830.

Sevitt, S. (1959), Modern trends in Accident Surgery and Medicine p. 247. London, Butterworth.

Sevitt, S., and Gallagher, N. (1961), *Brit. J. Surgery,* **48,** 475.

